# Leveraging Digital Assessments and Speech Analytics for Monitoring Changes in Sleep and Cognition

**DOI:** 10.1093/geroni/igaf122.310

**Published:** 2025-12-31

**Authors:** Shifali Singh

**Affiliations:** McLean Hospital, Boston, Massachusetts, United States

## Abstract

Tracking everyday cognitive functioning, sleep, and mood, beyond a single timepoint of assessment, is essential for understanding the progression of cognitive impairment and improving early diagnosis and treatment of disorders like AD/ADRDs. In this talk, we will explore the integration of digital cognitive assessments and speech analytics, focusing on how these tools can track intraindividual and interindividual variability in sleep and cognitive performance over time. We will present our approach, developed in collaboration between IBM and McLean Hospital/Harvard Medical School, which combines traditional neuropsychological evaluations with cutting-edge speech analytics powered by AI, machine learning (ML), and large language models (LLMs). By leveraging digital cognitive assessments, we capture cognitive and sleep fluctuations through daily prompts and testing, allowing for a more dynamic understanding of cognitive health. In addition, speech data collected through open-ended prompts and summaries are analyzed using advanced ML algorithms, including Whisper from OpenAI for acoustic analysis, and chatGPT for result interpretation, to detect subtle changes in speech characteristics that correlate with cognitive and sleep changes. This talk will highlight how our tools track cognitive and sleep fluctuations over time, focusing on intraindividual variability—how an individual’s sleep impacts their cognitive changes from day to day—and interindividual variability—how these changes compare across different individuals. By integrating biometric data from actigraphy monitors, such as heart rate and activity levels, we will discuss how one can further refine models to better characterize cognitive states and identify how sleep can be used to detect early signs of cognitive decline.

